# Comparative Meta-Analysis of Survival, Risk, and Treatment Efficacy in Immunotherapy for Metastatic Melanoma Using Random-, Fixed-, and Mixed-Effects Models

**DOI:** 10.3390/jcm14145017

**Published:** 2025-07-15

**Authors:** Jelena Ivetić, Jovana Dedeić, Srđan Milićević, Katarina Vidojević, Marija Delić

**Affiliations:** Faculty of Technical Sciences, University of Novi Sad, 21000 Novi Sad, Serbia; jelenaivetic@uns.ac.rs (J.I.); radenovicj@uns.ac.rs (J.D.); srdjan88@uns.ac.rs (S.M.); vidojevic9@uns.ac.rs (K.V.)

**Keywords:** meta-analysis, fixed-effects model, random-effects model, generalized linear mixed model, immune checkpoint inhibitors, melanoma immunotherapy, immune-related adverse events, overall survival, progression-free survival, therapeutic risk, clinical benefit

## Abstract

**Background:** Immune checkpoint inhibitors (ICIs) have reshaped the treatment landscape of metastatic melanoma. While combination regimens often demonstrate improved response and survival compared to monotherapy, they are also associated with a higher incidence of immune-related adverse events (irAEs). Understanding the balance between benefit and risk is essential for making informed treatment decisions, especially given the variability in reported outcomes across clinical trials. **Methods:** We conducted a systematic review and meta-analysis of 14 clinical trials (comprising 22 treatment arms and >5000 patients) comparing ICI monotherapy (nivolumab, ipilimumab, or pembrolizumab) and combination therapy (nivolumab + ipilimumab) in advanced melanoma. Treatment-related outcomes were synthesized using fixed-effects, random-effects, or generalized linear mixed models (GLMMs), depending on study variability. Survival data were extracted from published Kaplan–Meier curves and analyzed using longitudinal GLMMs to capture trends over time. **Results:** Compared to monotherapy, combination immunotherapy achieved higher clinical benefit, with an overall response of 52.2% (vs. 31.6%), a five-year overall survival of 55.7% (vs. 34.3%), and a five-year progression-free survival of 39.0% (vs. 17.2%). However, this benefit came with a higher risk of toxicity: immune-related adverse events occurred in 93.2% of patients receiving combination therapy versus in 81.9% receiving monotherapy. Differences were consistent across all individual severe toxicities. **Conclusions:** Combination immunotherapy offers greater long-term clinical benefit than monotherapy in metastatic melanoma but at the cost of increased toxicity. By applying models adapted to study variability, we provide more reliable estimates of treatment efficacy and risk. GLMMs provide the most robust estimates and enable the modeling of survival dynamics over time. These findings support evidence-based decision-making and highlight the value of model-informed meta-analysis in oncology.

## 1. Introduction

Immune checkpoint inhibitors (ICIs) have significantly transformed the treatment of metastatic melanoma, offering markedly improved survival compared to conventional chemotherapy, which provides a median overall survival (OS) of 4–12 months [1,2,3]. Multiple clinical trials have confirmed the benefit of both monotherapy and combination therapy, with durable outcomes over extended follow-up periods [1,2,4]. However, survival outcomes vary across studies, influenced by differences in patient populations, treatment protocols, and follow-up durations [2]. For example, the CheckMate 066 study reported a 39% OS rate for nivolumab versus 17% for dacarbazine [4]. The CheckMate 067 trial showed that combination therapy outperformed monotherapy, with a median OS of 72.1 months, compared to 36.9 months for nivolumab and 19.9 months for ipilimumab; progression-free survival (PFS) followed a similar pattern: 11.5, 6.9, and 2.9 months, respectively [2]. Notably, 77% of patients treated with the combination and 69% treated with nivolumab were treatment-free at 6.5 years [2], underscoring the potential for durable remission.

Despite these encouraging outcomes, ICIs are associated with a substantial risk of immune-related adverse events (irAEs), where immune responses target healthy tissue. Toxicities such as pneumonitis, colitis, thyroiditis, and pancreatitis may significantly impact quality of life [5,6,7]. Hribernik et al. [8] observed irAEs in 63% of patients on pembrolizumab, with 8.7% experiencing grade 3 or 4 events and 11.6% discontinuing treatment. Similarly, Robert et al. reported an 80.7% irAE rate, including 17.7% with grade 3 or 4 events and 8.8% with discontinuations [9]. In a pivotal trial, 86% of patients receiving monotherapy and 96% receiving combination therapy experienced irAEs [1,2], with grade 3 or 4 events occurring in 28%, 21%, and 59% of patients on ipilimumab, nivolumab, and their combination, respectively; the discontinuation rates were 16%, 12%, and 39%. Such variability highlights the challenges in synthesizing safety data across treatment regimens [8,9,10].

Balancing the therapeutic efficacy of ICIs with their risk of severe side effects requires a systematic approach. A meta-analysis offers a rigorous framework for integrating evidence from diverse clinical trials to support informed decision-making. By combining results from independent studies, a meta-analysis enhances statistical power and precision in estimating treatment effects [11,12,13], being particularly valuable in immunotherapy research, where study outcomes often differ. It also allows for estimating pooled confidence intervals (CIs) and identifying heterogeneity in treatment effects [13].

The robustness of a meta-analysis depends on how it addresses between-study differences. Fixed-effects models (FEMs) and random-effects models (REMs) are commonly used [11,12,14], with FEMs assuming a shared treatment effect and REMs allowing variability. However, these models may struggle with complex data typical of immunotherapy studies, such as uneven follow-ups or non-normally distributed outcomes. To overcome such limitations, generalized linear mixed models (GLMMs) have been introduced [15,16,17]. GLMMs are especially effective in modeling binary outcomes, survival data, and clustered structures, accommodating study design diversity and providing more robust, clinically relevant summaries in immunotherapy research.

Recent meta-analyses have investigated moderators of immune checkpoint inhibitor efficacy, including sex-based differences [18], the circadian timing of ICI administration [19], clinical variables in resectable non-small-cell lung cancer [20,21], and survival outcomes reconstructed from individual patient data in advanced mucosal melanoma [22]. While these studies have substantially advanced our understanding of treatment-specific effects within defined populations, a comprehensive, model-based comparison of survival, risk, and treatment efficacy outcomes in advanced melanoma remains lacking. The present study aims to address this gap.

In this study, our primary objective is to synthesize and compare the therapeutic profiles of immune checkpoint inhibitor (ICI) monotherapy and combination therapy in patients with advanced melanoma while properly addressing differences across clinical trials. Our analysis focuses on two key outcome categories relevant to treatment decisions: adverse events and survival.

First, for binary outcomes, such as the frequency of immune-related adverse events and response rates, we estimate pooled effect sizes using three statistical approaches: fixed-effects, random-effects, and generalized linear mixed models. This enables a direct comparison of the risks and benefits across treatment types while quantifying uncertainty within each model framework.

Second, for time-dependent outcomes, specifically progression-free survival (PFS) and overall survival (OS), we extract survival data from published Kaplan–Meier curves at multiple time points. These data are analyzed using a generalized linear mixed model approach, which allows us to evaluate how survival trends evolve over time while accounting for variation both within and between studies.

Rather than comparing statistical models directly, we match each modeling approach to the structure of the data, prevalence versus survival, ensuring both statistical validity and clinical interpretability. This strategy provides a more nuanced understanding of treatment effects and supports evidence-based decision-making in immunotherapy for melanoma. To the best of our knowledge, this is the first meta-analysis of metastatic melanoma to jointly model efficacy and toxicity using complementary statistical frameworks and time-resolved survival data. By integrating heterogeneous evidence through robust and clinically aligned modeling strategies, our study establishes a generalizable template for future analyses in oncology where time-to-event outcomes and between-study variation are central.

The remainder of this paper is organized as follows: Section 2 details the materials and methods, including study identification, data extraction, and the modeling strategies applied to both binary and longitudinal outcomes, as well as the influence of model choice and the rationale behind model selection. Section 3 presents the meta-analytic results for adverse events, therapeutic benefits, and survival outcomes, with emphasis on the influence of model choice. Section 4 discusses the methodological and clinical implications of our findings, including limitations and future directions for research in immunotherapy evidence synthesis. Section 5 includes the final remarks. Additional information on the included trials and extracted variables is provided in Appendix A.

## 2. Materials and Methods

### 2.1. Study Identification and Data Extraction

**Figure 1:**
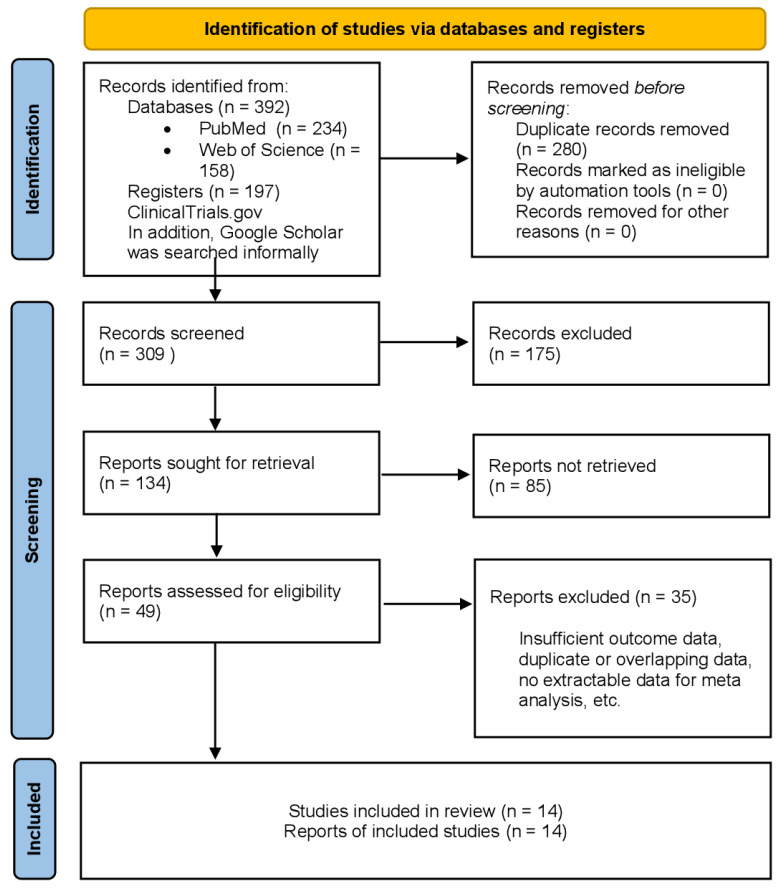
Flowchart of the systematic selection process of the studies included in this meta-analysis.

This meta-analysis systematically evaluated both the benefits and risks of immunotherapy in patients with advanced or metastatic melanoma. A systematic literature search was conducted in PubMed, Web of Science, Google Scholar, and ClinicalTrials.gov, covering publications up to December 2024. The search strategy combined terms such as "melanoma", "immune checkpoint inhibitors", "monotherapy", "combination therapy", "overall survival", "progression-free survival", and "immune-related adverse events". A flowchart illustrating the systematic selection process of the studies included in the meta-analysis is presented in Figure 1.

Studies were eligible if they met the following criteria:

- Involved adult patients with advanced or metastatic melanoma;
- Reported results for ICI monotherapy or a combination therapy;
- Provided extractable data on adverse events, clinical responses, or survival outcomes;
- Clearly separated results for monotherapy and combination therapy groups.

A total of 14 studies were included, contributing 22 different treatment arms (16 monotherapy and 6 combination therapy treatment arms). In this paper, monotherapy refers to treatment with a single ICI agent, nivolumab, ipilimumab, or pembrolizumab, whereas combination therapy involves regimens that include nivolumab plus ipilimumab; the nivolumab–relatlimab combination was not included. The authors participated in the literature search in an organized manner, following the same search principles to ensure consistency. The variables extracted included the study design, sample size, treatment details, adverse event rates, clinical response, and survival data.

Therapeutic benefits were assessed through variables including complete response (CR), partial response (PR), overall response (OR), and stable disease (SD). Risks were examined by analyzing the incidence of any grade of immune-related adverse events (irAEs), with a focus on pneumonitis, colitis, diarrhea, increased aspartate aminotransferase (AST), increased alanine aminotransferase (ALT), and two forms of thyroiditis. As increased AST and ALT levels may indicate underlying immune-mediated hepatitis, a clinically relevant irAE, they were included as separate outcomes in the analysis. Survival outcomes were evaluated using overall survival (OS) and progression-free survival (PFS), extracted across time points from Kaplan–Meier curves. All analyzed data were obtained from the references listed in the bibliography [2,4,8,23,24,25,26,27,28,29,30,31,32,33,34] and briefly summarized in Appendix A, Table A1 and Table A2.

When survival data were not explicitly reported, values were digitized from Kaplan–Meier curves using WebPlotDigitizer software, v4.5 (Ankit Rohatgi, Pacifica, CA, USA) [35]. All proportions were recalculated if needed, and binomial approximations were used to derive 95% confidence intervals. This systematic review has not been registered.

### 2.2. Meta-Analysis of Adverse Events and Benefits

A central challenge in synthesizing findings from multiple clinical studies is addressing the heterogeneity arising from differences in study populations, treatment protocols, and methodological designs. To account for this variability, we employed a stepwise meta-analytic framework incorporating a fixed-effects model (FEM), a random-effects model (REM), and a generalized linear mixed model (GLMM), allowing us to assess treatment effects under varying assumptions about inter-study variability. All outcomes—including the pooled prevalence of immune-related adverse events (irAEs) and clinical benefit rates—were evaluated separately for monotherapy and combination therapy arms, using the model best suited to the observed heterogeneity.

Heterogeneity was assessed using Cochran’s *Q* statistic and the $I^{2}$ statistic, which jointly capture the presence and magnitude of variability beyond chance. Between-study variance ($\tau^{2}$) was estimated using the DerSimonian–Laird (DL) method, a widely applied, non-iterative approach valued for its simplicity and robustness in accounting for inter-study differences [13,14].

#### 2.2.1. Heterogeneity Assessment and Model Selection

Cochran’s *Q* statistic serves as a diagnostic test for the presence of heterogeneity between study arms. It is defined as follows:

(1) $$Q=\sum_{i=1}^{k}w_{i}{\left(p_{i}-\bar{p}\right)}^{2},$$

where k≥2 is the number of study arms, $p_{i}$ is the proportion of events observed in the study arm i∈{1,2,…,k}, and

(2) $$w_{i}=\frac{1}{\sigma_{i}^{2}}$$

represents the inverse-variance weight. Here, $\sigma_{i}^{2}$ denotes the estimated sampling variance for study arm *i*, which is commonly approximated under a binomial model as follows:

(3) $$\sigma_{i}^{2}=\frac{p_{i}\left(1-p_{i}\right)}{n_{i}},$$

where $p_{i}$ is the observed event proportion, and $n_{i}$ is the sample size in study arm *i*. The estimate of the pooled fixed effects, denoted by $\bar{p}$, is as follows:

(4) $$\bar{p}=\frac{\sum_{i=1}^{k}w_{i}p_{i}}{\sum_{i=1}^{k}w_{i}}.$$

To interpret the degree of heterogeneity, we calculated the $I^{2}$ statistic:

(5) $$I^{2}=\max\left(0,\frac{Q-(k-1)}{Q}\right)\times 100\%.$$

The $I^{2}$ statistic quantifies the proportion of total variation across study arms that is due to heterogeneity rather than chance, offering a practical measure for evaluating consistency in meta-analysis results. According to [36], $I^{2}$ values lower than 25% may be interpreted as indicating low heterogeneity, 50% may be interpreted as moderate, and 75% or higher may be interpreted as substantial to considerable heterogeneity. Following this framework, we adopted a conservative modeling strategy based on the heterogeneity magnitude.

Specifically, an FEM was applied when $I^{2}<25\%$, indicating low variability among studies. For $25\%\leq I^{2}<90\%$, an REM was employed to account for moderate to substantial between-study variance. When heterogeneity reached extreme levels ($I^{2}\geq 90\%$), we utilized a GLMM, which has been recommended in high-variance contexts such as prevalence and proportion meta-analyses [37]. This structured model selection aimed to better align statistical complexity with the level of heterogeneity.

While $I^{2}$ values exceeding 75% are considered to reflect considerable heterogeneity [36], we adopted a more stringent threshold of 90% when switching to a GLMM. This decision reflects emerging evidence from recent methodological studies indicating that conventional random-effects models often become unstable when $I^{2}$ surpasses 90%, particularly in the context of meta-analyses of proportions and prevalence. In such scenarios, a GLMM offers increased modeling robustness by working on transformed (e.g., logit) scales and by explicitly accounting for random intercepts and non-constant variance structures.

#### 2.2.2. Fixed-Effects Model (FEM)

Under the FEM framework, it is assumed that all included studies estimate the same underlying effect size, meaning that the true proportion of events is identical across all studies.

The pooled estimate of the proportion ${\bar{p}}_{\mathrm{FEM}}$ is calculated as a weighted average of study-specific estimates, with weights determined by the inverse of the variance. The formula for the pooled estimate of the proportion is as follows:

(6) $$\begin{array}{cc} {\bar{p}}_{\mathrm{FEM}} & =\frac{\sum_{i=1}^{k}w_{i}p_{i}}{\sum_{i=1}^{k}w_{i}}, \end{array}$$

which corresponds to $\bar{p}$ from Equation (4).

The variance of the pooled estimate of the proportion is given by

(7) $$\begin{array}{cc} \mathrm{Var}({\bar{p}}_{\mathrm{FEM}}) & =\frac{1}{\sum_{i=1}^{k}w_{i}}, \end{array}$$

and the standard error is

(8) $$\begin{array}{c} {\mathrm{SE}}_{\mathrm{FEM}}=\sqrt{\mathrm{Var}({\bar{p}}_{\mathrm{FEM}})}. \end{array}$$

Based on this standard error, a 95% confidence interval (CI) for the pooled estimate of the proportion under the FEM is constructed as follows:

(9) $$\begin{array}{c} ({\bar{p}}_{\mathrm{FEM}}-1.96\cdot {\mathrm{SE}}_{\mathrm{FEM}},{\bar{p}}_{\mathrm{FEM}}+1.96\cdot {\mathrm{SE}}_{\mathrm{FEM}}). \end{array}$$

An FEM is most suitable when clinical, methodological, and statistical heterogeneity are minimal or absent. In such settings, an FEM offers more precise estimates due to narrower confidence intervals and is often favored in meta-analyses where study protocols and populations are tightly controlled, such as in oncology. Its simplicity and efficiency make it a logical choice when study conditions are standardized, particularly in early-phase or protocol-driven trials. However, an FEM assumes a common true effect and ignores between-study variability, which can lead to underestimated uncertainty when heterogeneity is present.

#### 2.2.3. Random-Effects Model (REM)

When moderate heterogeneity was present, which means Q>k−1, we proceeded to estimate the variance $\tau^{2}$ between the study arms using the DerSimonian–Laird method:

(10) $$\tau^{2}=\frac{Q-(k-1)}{C},$$

where

(11) $$C=\sum_{i=1}^{k}w_{i}-\frac{\sum_{i=1}^{k}w_{i}^{2}}{\sum_{i=1}^{k}w_{i}}.$$

This value quantifies the variance in the true effect sizes between study arms.

The REM accounts for this by modifying the weight of each study arm:

(12) $$w_{i}^{*}=\frac{1}{\sigma_{i}^{2}+\tau^{2}}.$$

The pooled estimate under the REM is then

(13) $${\bar{p}}_{\mathrm{REM}}=\frac{\sum_{i=1}^{k}w_{i}^{*}p_{i}}{\sum_{i=1}^{k}w_{i}^{*}}.$$

The variance of the pooled estimate of the proportion is given by

(14) $$\begin{array}{cc} \mathrm{Var}({\bar{p}}_{\mathrm{REM}}) & =\frac{1}{\sum_{i=1}^{k}w_{i}^{*}}, \end{array}$$

and the standard error is

(15) $${\mathrm{SE}}_{\mathrm{REM}}=\sqrt{\mathrm{Var}({\bar{p}}_{\mathrm{REM}})}.$$

Based on this standard error, a 95% CI for the pooled estimate of the proportion under the REM is constructed as follows:

(16) $$({\bar{p}}_{\mathrm{REM}}-1.96\cdot {\mathrm{SE}}_{\mathrm{REM}},{\bar{p}}_{\mathrm{REM}}+1.96\cdot {\mathrm{SE}}_{\mathrm{REM}}).$$

This methodology provides a flexible and statistically grounded approach to synthesizing evidence when treatment effects vary between study arms, a common occurrence in immunotherapy trials due to diverse patient populations, study protocols, and endpoints.

The decision to employ an REM is guided by both statistical diagnostics and a clinical understanding of immune-related variability. In the context of ICI treatments, individual studies often report wide-ranging outcomes due to differences in tumor burden, immune profiles, and previous therapies. Consequently, an REM not only offers statistical rigor but also better reflects real-world clinical diversity.

#### 2.2.4. Generalized Linear Mixed Model (GLMM)

To capture between-study arm variability in cases of extreme heterogeneity, we implemented a GLMM applied to logit-transformed event proportions $p_{i}$ for study arm *i*. In cases of extreme heterogeneity ($I^{2}\geq 90\%$), a GLMM provides a more robust alternative to conventional random-effects models [16]. The transformed variable is

(17) $$y_{i}=\ln\left(\frac{p_{i}}{1-p_{i}}\right),$$

and the model equation is

(18) $$y_{i}=\beta_{0}+u_{i},$$

where $\beta_{0}$ is the intercept coefficient, and $u_{i}∼N\left(0,\tau^{2}\right)$ is the random intercept for study arm *i*, where $\tau^{2}$ is the between-study arm variance from Equation (10).

After fitting the model parameters with a logit link, predicted values ${\hat{y}}_{i}$ are obtained on the logit scale. These values are transformed back to the original proportion scale using the inverse logit function:

(19) $${\hat{p}}_{i}={\mathrm{logit}}^{-1}\left({\hat{y}}_{i}\right)=\frac{e^{{\hat{y}}_{i}}}{1+e^{{\hat{y}}_{i}}}.$$

The same inverse logit transformation is applied to the lower and upper bounds of the 95% confidence intervals on the logit scale, denoted as ${\hat{y}}_{i}^{L}$ and ${\hat{y}}_{i}^{U}$, respectively:

(20) $$\left(\frac{e^{{\hat{y}}_{i}^{L}}}{1+e^{{\hat{y}}_{i}^{L}}},\;\frac{e^{{\hat{y}}_{i}^{U}}}{1+e^{{\hat{y}}_{i}^{U}}}\right).$$

This ensures that both point estimates and confidence intervals are interpretable on the original proportion scale.

The model used in this analysis relies on the Lmer function from the pymer4 Python package, which provides access to linear mixed models (LMMs) and generalized linear mixed models (GLMMs) by interfacing with the lme4 package implemented in R.

For each pooled proportion estimate derived from the respective models (Equations (6), (13), and (19)), we performed a two-proportion Z-test to assess the differences between monotherapy and combination therapy:

(21) $$Z=\frac{{\bar{p}}_{1}-{\bar{p}}_{2}}{\sqrt{{\mathrm{SE}}_{1}^{2}+{\mathrm{SE}}_{2}^{2}}},$$

where ${\bar{p}}_{1}$ and ${\bar{p}}_{2}$ are the pooled estimates for each group, and ${\mathrm{SE}}_{1}$ and ${\mathrm{SE}}_{2}$ are their standard errors, respectively.

Forest plots and summary charts were used to visualize the pooled proportions and their respective confidence intervals for both risk and benefit outcomes.

### 2.3. Modeling of Overall and Progression-Free Survival

The analysis included 14 published studies, from which 22 distinct treatment arms were defined based on monotherapy or combination immunotherapy groups. Of these, 19 arms reported Kaplan–Meier (KM) survival data suitable for extraction. Time-specific overall survival (OS) proportions were extracted from the published KM curves using WebPlotDigitizer software, v4.5 [35]. For each treatment arm, survival percentages were manually digitized at standard time intervals (typically 12, 24, 36, 48, and 60 months), depending on the reporting granularity of each study. The corresponding number of patients at risk at baseline was obtained from study tables or figure captions. This process yielded a total of 66 time point-level OS observations across the 19 KM-reported arms.

In parallel, progression-free survival (PFS) data were extracted from 16 KM-reported arms using the same methodology, yielding 60 time point-level PFS observations. Both OS and PFS datasets were used to construct a GLMM designed to evaluate longitudinal trends in survival outcomes over time. The model included time (in months), therapy type (monotherapy or combination therapy), and their interaction as fixed effects, with treatment arm specified as the grouping factor and modeled as a random intercept.

The extracted OS and PFS percentages were converted into proportions $p_{ij}$, representing the estimated probability of survival at time $t_{j}$ for study arm *i*. To stabilize variance and allow for linear modeling, the proportions were transformed using the logit function:

(22) $$y_{ij}=\ln\left(\frac{p_{ij}}{1-p_{ij}}\right).$$

The binomial standard error for each transformed proportion was approximated as follows:

(23) $$\mathrm{SE}\left(y_{ij}\right)=\sqrt{\frac{1}{n_{ij}\cdot p_{ij}\cdot \left(1-p_{ij}\right)}},$$

where $n_{ij}$ is the number of patients in study *i* contributing to the estimate at time $t_{j}$. The proportions were truncated to the [0.01, 0.99] interval to avoid infinite logits.

A GLMM was then fitted to the logit-transformed OS and PFS proportions using restricted maximum likelihood estimation (REML). The model included the following:

- A fixed effect for time (in months);
- A fixed effect for therapy type (coded as 0 = combination and 1 = monotherapy);
- An interaction term between time and therapy type;
- A random intercept for study ID to account for between-study variability.

The model equation is

(24) $$y_{ij}=\beta_{0}+\beta_{1}\cdot {\mathrm{TT}}_{i}+\beta_{2}\cdot t_{j}+\beta_{3}\cdot \left({\mathrm{TT}}_{i}\cdot t_{j}\right)+u_{i}+\varepsilon_{ij},$$

where:

- $y_{ij}$ is the logit-transformed OS or PFS at time $t_{j}$ for study arm *i*;
- ${\mathrm{TT}}_{i}\in \left\{0,1\right\}$ indicates therapy type (combination therapy or monotherapy);
- $u_{i}$ is the zero-mean normally distributed random intercept per study arm;
- $\varepsilon_{ij}$ is the zero-mean normally distributed residual error.

Model parameters were estimated using the statsmodels library in Python, v0.14, (Python Software Foundation, Wilmington, DE, USA). Predictions were back-transformed using the inverse logit function to provide interpretable survival percentages at selected time points. Confidence intervals were calculated on the logit scale using the delta method, based on the full covariance matrix of the fixed effects, and they were subsequently transformed to the probability scale. The GLMM framework was selected based on established methods for modeling proportion data with within-study clustering [15,16,17].

It is important to note that our GLMM-based survival analysis does not model time-to-event data in the traditional sense, nor does it incorporate censoring. Instead, we extract discrete survival proportions from published KM curves at multiple clinically relevant time points, and we model these logit-transformed values using generalized linear mixed models. This approach enables a meta-analysis of longitudinal survival trends while accounting for study-level heterogeneity via random intercepts. While it cannot reconstruct full survival functions or hazard rates, this method allows for consistent comparisons across treatment arms in the absence of individual patient data.

### 2.4. Influence of Model Choice and Rationale for Model Selection

Here, we provide a detailed examination of the three statistical models used in the analysis, aiming to clarify the rationale behind their selection, as well as the specific advantages and limitations that each model presents in different contexts. Particular attention is given to the assumptions that these models make regarding heterogeneity across studies and their ability to accommodate data structures typical of clinical research. One of the central challenges in synthesizing evidence from multiple clinical studies lies in managing heterogeneity stemming from differences in study populations, treatment protocols, and methodological approaches. To address this, we applied a stepwise meta-analytic strategy that integrates three models: an FEM, an REM, and a GLMM. This framework enabled us to explore treatment effects under different assumptions regarding variability between studies and the structure of the underlying data.

The FEM assumes that all included studies estimate the same underlying treatment effect and is most appropriate when heterogeneity is minimal. In contrast, the REM introduces a between-study variance component, yielding more conservative confidence intervals and making it a better fit in the presence of moderate heterogeneity. However, it has been shown that traditional inverse-variance estimators of $\tau^{2}$ and the overall effect can be biased, particularly in the context of odds ratios. The study in [38] proposed improved methods for $\tau^{2}$ estimation and overall effect inference, demonstrating in simulation studies that standard REM approaches often yield suboptimal coverage and biased estimates. The GLMM builds on these approaches by incorporating study-specific random effects and directly modeling binary outcomes within a hierarchical structure. As such, the GLMM is particularly well suited for meta-analyses that involve unbalanced study designs, variations in sample sizes, or differing event rates across trials [39].

We assessed all outcomes, including the pooled prevalence of irAEs and clinical benefit rates, separately for monotherapy and combination therapy groups using the most appropriate model, according to the heterogeneity analysis. While the FEM produced the narrowest confidence intervals, it tended to underestimate uncertainty when heterogeneity was present. The REM offered improved interval coverage but was more sensitive to small-study effects. Among the three, the GLMM showed the greatest robustness in estimating both effect sizes and variance components, particularly in settings with substantial design and outcome variability.

In practical terms, the FEM may be appropriate when studies are highly comparable in design and population characteristics. The REM is more suitable when moderate heterogeneity is expected and a sufficient number of studies is available. However, in fields such as oncology where heterogeneity in patient populations, study designs, and outcome measures is common, the GLMM provides a more flexible and reliable framework for inference and is generally the preferred choice. Nevertheless, even the GLMM relies on assumptions that may be restrictive in some applications. For example, time-invariant latent variables may influence outcomes differently across time or interact variably with observed covariates.

## 3. Results

### 3.1. Adverse Events and Benefit Outcomes

**Figure 2:**
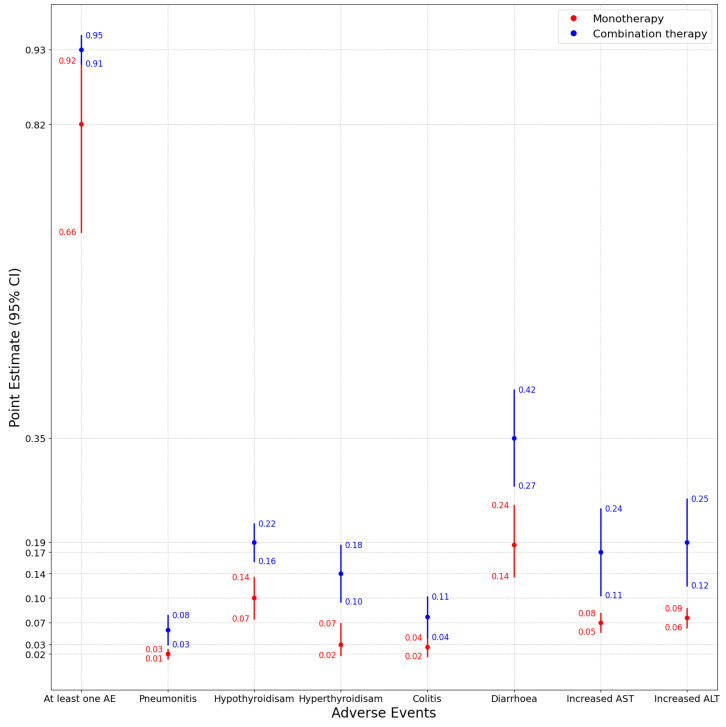
Forest plot showing point estimates with 95% confidence intervals for adverse events under monotherapy and combination therapy.

**Figure 3:**
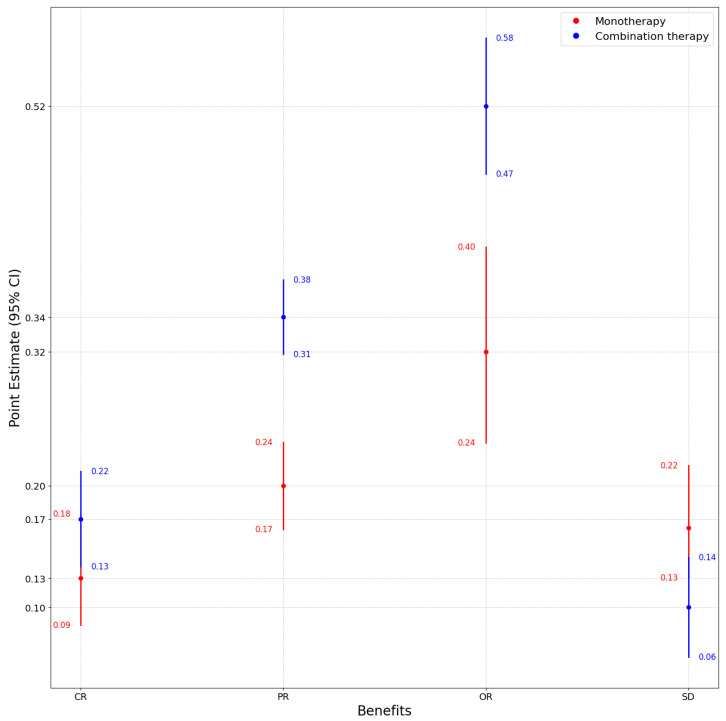
Forest plot showing point estimates with 95% confidence intervals for therapy benefits under monotherapy and combination therapy.

We assessed the prevalence of adverse events (AEs) and clinical benefit outcomes separately for monotherapy and combination immunotherapy treatment arms by using a model selection strategy based on between-study arm heterogeneity. Summaries of the model choice, pooled estimates, and statistical comparison are presented in Table 1 and Table 2 and Figure 2 and Figure 3.

#### 3.1.1. Results for Adverse Events

The prevalence of experiencing at least one AE was significantly higher in the combination therapy group (0.9322, 95% CI: (0.9111, 0.9534)) than in the monotherapy group (0.8194, 95% CI: (0.6562, 0.9152)), with a highly significant difference (p<0.001). Due to substantial heterogeneity across monotherapy studies ($I^{2}=99.53\%$), we applied a GLMM, whereas the combination therapy group used an REM due to moderate heterogeneity ($I^{2}=61.50\%$).

For specific irAEs, pneumonitis occurred in 5.55% of patients receiving combination therapy and 1.95% receiving monotherapy, both modeled via the REM. Hypothyroidism was less prevalent in monotherapy (10.41%, REM) than in combination therapy (18.78%, REM), while hyperthyroidism showed a similar trend, being higher in the combination therapy group (14.09% vs. 3.39%). Colitis and diarrhea were also more frequent with combination therapy (7.53% and 34.57%, respectively), with all differences being statistically significant (p<0.001). The incidence of increased AST and ALT was likewise higher with combination therapy (17.32% and 18.81%, respectively) than with monotherapy (6.70% and 7.38%, respectively).

In addition to statistical significance, we evaluated the magnitude and uncertainty of the observed differences in adverse event prevalence between monotherapy and combination therapy. For the incidence of any-grade adverse events, the absolute effect size reached 11.3% (93.2% vs. 81.9%), with narrowly overlapping 95% confidence intervals (CI: 91.1–95.3% vs. 65.6–91.5%), suggesting both statistical and clinical relevance. Across individual immune-related adverse events (irAEs), the absolute effect sizes between the combination and monotherapy arms ranged from a minimal difference of 3.60% for pneumonitis (5.55% vs. 1.95%) to a maximum of 16.12% for diarrhea (34.57% vs. 18.45%). The confidence intervals were non-overlapping for all outcomes. Notably, the narrowest confidence intervals were observed for low-heterogeneity variables with low frequency, including pneumonitis (modeled using the REM), increased AST, and increased ALT (both modeled using the FEM), all in the monotherapy group—reflecting lower between-study variability.

#### 3.1.2. Results for Clinical Benefits

Across most of the evaluated benefit outcomes, combination therapy outperformed monotherapy. The complete response (CR) was 17.49% (REM) vs. 12.51% (GLMM), while the partial response (PR) reached 34.48% (FEM) vs. 20.29% (REM). The overall response (OR) was significantly higher in the combination therapy group (52.24% vs. 31.56%), while monotherapy had a higher pooled proportion of stable disease (16.76%). All differences between groups were significant at p<0.001.

An evaluation of effect sizes across clinical benefit outcomes revealed substantial differences between treatment modalities. Absolute differences ranged from 4.98% for complete response (CR) to 20.68% for overall response (OR), with the largest gain observed in favor of combination therapy. Partial response (PR) also exhibited a notable increase of 14.19%. In contrast, stable disease (SD) was more common in the monotherapy arms by 6.69%, reflecting a potential shift from disease control to active tumor regression under combination regimens. The confidence intervals for PR and OR were non-overlapping, reinforcing the statistical robustness of the observed differences. Notably, the narrowest confidence interval was seen for PR in the combination group (CI: 31.35%–37.61%), modeled using the FEM due to small between-study heterogeneity.

### 3.2. OS and PFS Outcomes

In addition to the pooled proportions of risk and response outcomes, we performed a longitudinal meta-analysis of survival endpoints to assess the temporal dynamics of treatment efficacy. Specifically, we analyzed overall survival (OS) and progression-free survival (PFS) across time points extracted from KM curves, using a GLMM to account for within-study clustering and between-study heterogeneity. The results are presented separately for the monotherapy and combination immunotherapy arms.

#### 3.2.1. Results for Overall Survival (OS)

A GLMM was used to evaluate the longitudinal trend in OS across multiple time points for patients treated with monotherapy versus combination immunotherapy. The model results, presented in Table 3, indicate that combination therapy was associated with a significantly higher baseline OS compared to monotherapy (logit difference = −0.639, p<0.001). OS decreased over time in both groups (time effect: $\beta_{2}=-0.014$, p<0.001), but the rate of decline was steeper in the monotherapy group (time × therapy interaction: $\beta_{3}=-0.004$, p=0.253). These findings suggest that combination immunotherapy not only confers a survival advantage at baseline but also maintains a more gradual decline in OS over time.

**Figure 4:**
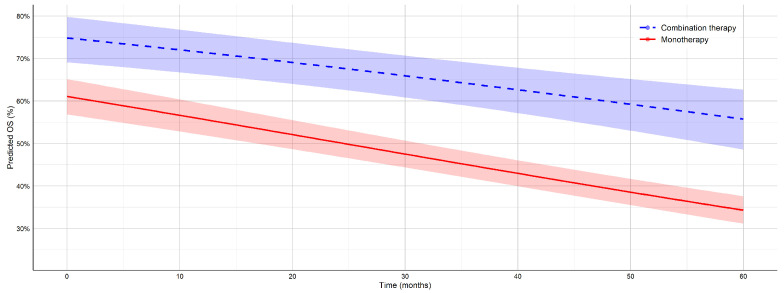
Predicted trends of OS over time for monotherapy and combination therapy in patients with melanoma, with associated 95% confidence intervals. Estimates are based on a GLMM using logit-transformed survival proportions.

The predicted OS estimates at clinically relevant time points are summarized in Table 4. At 12 months, the model estimates an OS of 71.5% (95% CI: 66.2–76.2%) for combination therapy, compared to 55.7% (95% CI: 52.0–59.4%) for monotherapy. This gap persists and widens slightly over time; at 60 months, the predicted OS for combination therapy is 55.7% (95% CI: 48.6–62.7%) versus 34.3% (95% CI: 31.1–37.6%) for monotherapy. These model-based projections further support the advantage of combination immunotherapy in maintaining long-term survival. The predicted trends of OS over time for monotherapy and combination therapy are shown in Figure 4.

#### 3.2.2. Results for Progression-Free Survival (PFS)

A GLMM was fitted to evaluate the longitudinal PFS trend across multiple time points for patients treated with monotherapy versus combination immunotherapy. The model was fitted on 60 observations from 16 KM-extracted arms. Time (in months), therapy type (monotherapy vs. combination), and their interaction were included as fixed effects, with a random intercept specified for study ID.

The model results, summarized in Table 5, show that combination therapy was associated with a significantly higher baseline PFS compared to monotherapy (logit difference = −0.776, p=0.003). PFS decreased significantly over time in both groups (time effect: $\beta_{2}=-0.008$, p=0.020), with a steeper decline in the monotherapy group (time × therapy interaction: $\beta_{3}=-0.006$, p=0.125), indicating a marginally significant differential trend.

**Figure 5:**
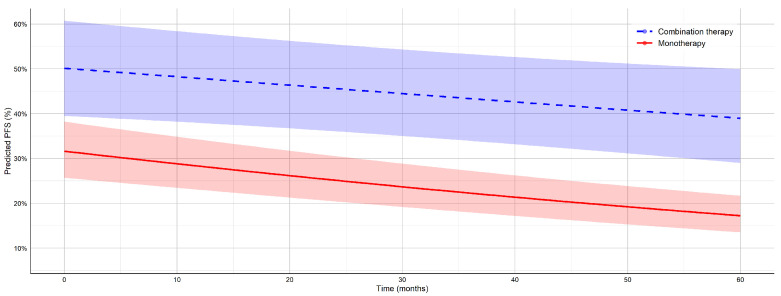
Predicted trends of PFS over time for monotherapy and combination therapy in patients with melanoma, with associated 95% confidence intervals. Estimates are based on a GLMM using logit-transformed survival proportions.

The predicted PFS estimates at clinically relevant time points are summarized in Table 6. At 12 months, the model estimates a PFS of 47.9% (95% CI: 37.9–58.0%) for combination therapy, compared to 28.3% (95% CI: 23.0–34.2%) for monotherapy. This disparity continues to grow over time; at 60 months, the predicted PFS for combination therapy is 39.0% (95% CI: 29.0–50.0%) versus 17.2% (95% CI: 13.5–21.7%) for monotherapy. These model-based projections further support the sustained benefit of combination immunotherapy in delaying disease progression in the long term. Figure 5 illustrates these predicted PFS trajectories with associated 95% confidence intervals for both treatment modalities.

A comparison between OS and PFS outcomes reveals both consistent and divergent trends across treatment modalities. For both endpoints, combination immunotherapy demonstrates a clear advantage over monotherapy, reflected in higher predicted survival estimates at all time points. The steeper decline observed in the monotherapy arms for OS compared to PFS may indicate earlier therapeutic resistance or disease relapse in the absence of synergistic immune modulation. Together, these findings reinforce the clinical rationale for selecting combination immunotherapy for long-term disease control.

Across both OS and PFS endpoints, model-based predictions revealed sustained and clinically meaningful survival advantages for combination therapy over monotherapy. At 60 months, the absolute effect size for OS reached 21.4% (55.7% vs. 34.3%), and that for PFS reached 21.8% (39.0% vs. 17.2%). These differences remained stable across all time points, with non-overlapping confidence intervals consistently favoring combination therapy. Although the time-by-therapy interaction term did not reach statistical significance, the divergence in the predicted survival trajectories suggests a progressively widening gap in long-term benefits. The width of the confidence intervals remained within acceptable bounds across time points, indicating adequate model stability and estimate precision.

## 4. Discussion

Our results underscore the methodological value of adapting model complexity to data heterogeneity. For example, PFS and OS were best modeled using a GLMM, as they involve longitudinal proportions influenced by latent clinical and temporal factors. Likewise, pooled estimates of adverse events and clinical benefits demonstrated that precise estimation and group comparison are possible even in highly variable datasets, provided that the correct statistical model is applied. While our findings confirm known clinical trends, the primary aim of this work was to highlight the analytical pathway that supports such inferences. All conclusions were derived via clearly defined probabilistic models, which were fit to appropriately transformed data and subjected to inferential rigor.

Numerous recent meta-analyses have examined the efficacy and safety of immune checkpoint inhibitors (ICIs) in oncology, applying diverse analytical frameworks and focusing on different moderators of treatment response. For instance, Conforti et al. [18] explored sex-based differences in ICI efficacy and found a significantly greater overall survival benefit in male patients (HR 0.72, 95% CI 0.65–0.79) than in females (HR 0.86, 95% CI 0.79–0.93). Landre et al. [19] explored novel moderators such as the time of day of ICI infusion, where a recent meta-analysis showed superior outcomes when ICIs were administered earlier in the day, suggesting that biological timing may modulate treatment response (OS HR 0.50, 95% CI 0.42–0.58; PFS HR 0.51, 95% CI 0.42–0.61), possibly reflecting circadian influences on immune function. Moreover, a comprehensive meta-analysis [20] in resectable non-small-cell lung cancer (NSCLC) assessed the impact of neoadjuvant chemoimmunotherapy versus chemotherapy across surgical, pathological, and efficacy endpoints. Importantly, even patients with low PD-L1 expression (<1%) demonstrated a significant benefit in event-free survival (HR 0.74, 95% CI 0.62–0.89), although no difference was observed in overall survival. Recently, Patel et al. [21] conducted a comparative meta-analysis of randomized trials on the overall survival of resectable non-small-cell lung cancer to assess the timing of immunotherapy (neoadjuvant, peri-operative, and postoperative), finding no statistically significant OS difference between timing groups, although neoadjuvant chemo-immunotherapy approaches appeared preferable due to a shorter treatment duration and lower costs. These findings underscore the need to account for multiple biological and treatment-related modifiers when assessing the efficacy of immunotherapy.

In contrast to these studies, which focused on specific populations or treatment conditions, our meta-analysis addresses survival and risk outcomes in metastatic melanoma across a range of clinical trials, applying a comparative model-based framework. We account for inter-study heterogeneity and assess the impact of model selection on pooled estimates. By situating our results alongside prior findings that examine sex, tumor biology, timing, and PD-L1 status as potential moderators, we extend the literature by offering a comprehensive, model-driven meta-analytic comparison focused on survival and risk outcomes within a single tumor type. This methodology not only enables a transparent synthesis of heterogeneous evidence but also offers a replicable analytic structure for future comparative meta-analyses in immuno-oncology. A key methodological contribution of our study is the application of a longitudinal generalized linear mixed model framework, which enables time-resolved modeling of survival outcomes. In contrast, previous meta-analyses primarily relied on point estimates or aggregate-level comparisons. For example, the Bayesian network meta-analysis by Silveira Nogueira Lima et al. [40] integrated evidence from immunotherapy and targeted therapy trials to estimate relative treatment rankings but did not capture survival dynamics over time. Teo et al. [22] reconstructed individual patient data from Kaplan–Meier curves, yet their analyses were based on a restricted mean survival time and a restricted mean time lost, without evaluating treatment trajectories. Elias et al. [41] performed subgroup analyses by age using aggregate data but did not incorporate longitudinal modeling. In contrast, our GLMM-based approach allows for the continuous modeling of OS and PFS over multiple time points, offering a more detailed view of treatment efficacy over time. Additionally, by jointly assessing both efficacy and toxicity using an FEM, an REM, and a GLMM, our analysis provides a more comprehensive assessment of the benefit–risk profile of immune checkpoint inhibitors in metastatic melanoma.

To enable a consistent comparison with the results of Teo et al.’s study [22], here, we focus on the 12-month OS rates reported in both studies. In our analysis, combination ICI therapy achieved a 12-month OS of 71.5% and a PFS of 47.9%, compared to 55.7% and 28.3% for monotherapy. These values closely align with those reported in a recent individual patient data meta-analysis of mucosal melanoma, which showed 12-month OS rates of 71.8% for combination therapy and 64.0% for monotherapy, as well as PFS rates of 35.1% and 28.3%, respectively. However, unlike [22], which was limited to short-term outcomes, our work also included long-term follow-up, demonstrating a 5-year OS of 55.7% and 34.3% and a PFS of 39% and 17.2% for combination therapy and monotherapy. These findings underscore the sustained benefit of combination immunotherapy in advanced melanoma and highlight the importance of long-term survival analyses in evaluating treatment efficacy. The observed differences may also be influenced by biological variation between cutaneous and mucosal melanoma subtypes, with cutaneous forms generally exhibiting a greater immunogenicity and response to ICI.

### Limitations and Future Directions

Several limitations of this meta-analysis should be acknowledged. First, the available data exhibited a notable imbalance in study representation between study arms, with a greater number of trials reporting outcomes for monotherapy compared to combination immunotherapy. This discrepancy may introduce asymmetry in the precision of pooled estimates and may affect the robustness of comparative analyses. Second, PFS and OS were not reported uniformly across studies, either in terms of the follow-up duration or the specific time points at which survival probabilities were extracted. While we addressed this by harmonizing data as proportions at available time points and applying a GLMM to account for study-level heterogeneity, the lack of standardized survival intervals limits the temporal comparability across studies. Third, the analysis of adverse events was constrained by the absence of temporal data on irAE onset. Reported AE rates were cumulative and did not indicate whether events occurred before, during, or after the time points used to assess OS. This temporal ambiguity constrains the interpretability of any analysis attempting to correlate toxicity with survival probability. Fourth, we grouped anti-PD-1 and anti-CTLA-4 agents under a single “monotherapy” category despite known mechanistic and toxicity differences. This decision was based on consistent adverse event patterns across monotherapy studies in our dataset, as well as prior literature that treated single-agent immune checkpoint inhibitors collectively [29,30]. While this grouping enabled broader comparisons with combination therapy, it may have obscured agent-specific safety signals and should be interpreted with caution. Fifth, we did not stratify analyses based on treatment line, as this information was inconsistently reported across studies. Although treatment setting can influence both efficacy and toxicity, introducing this criterion would have significantly reduced the number of eligible studies and compromised the statistical power of our comparisons. This limitation reflects the trade-off between methodological granularity and dataset comprehensiveness.

Finally, a methodological consideration in our survival analysis is the use of a GLMM to model survival proportions over time. This approach allowed us to perform a longitudinal meta-analysis across heterogeneous studies using aggregate data extracted from KM curves. While a GLMM does not incorporate right-censoring and relies on logit-transformed survival proportions rather than individual time-to-event data, it offers a flexible framework for capturing between-study variation and temporal trends. These features made it particularly suitable for the structure of our dataset, where individual-level data were unavailable. We acknowledge that a GLMM assumes smooth survival trajectories between time points and may be less suited to settings with strong time-varying hazards. However, within the context of our objectives and data availability, this method provided a robust and interpretable model for comparing survival outcomes.

Future work should prioritize access to individual patient-level data, which would enable time-dependent modeling strategies. Joint models—linking longitudinal AE occurrence with survival endpoints—could provide more accurate insights into whether early irAEs can serve as surrogate markers for therapeutic efficacy. These directions are increasingly supported in the literature, with recent studies using real-world data to examine the temporal association between immune-related toxicity and survival outcomes in melanoma immunotherapy [42,43,44]. In parallel, Bayesian frameworks may offer a flexible alternative to frequentist GLMMs for synthesizing sparse or heterogeneous clinical data [40]. These directions represent promising avenues for strengthening both the accuracy and interpretability of evidence in immunotherapy and beyond.

## 5. Final Remarks

This study demonstrates the application of formal meta-analytical modeling to assess the risk–benefit profile of immunotherapy regimens in melanoma treatment. By integrating multiple modeling strategies, ranging from classical fixed- and random-effects models to generalized linear mixed models, we were able to appropriately handle heterogeneous data structures and varying effect sizes across clinical endpoints. The use of a model selection procedure driven by heterogeneity (quantified via the $I^{2}$ statistic) enabled flexible and robust estimation across distinct outcome types. Specifically, an FEM was employed in homogeneous contexts, while REM and GLMM approaches captured both moderate and high between-study arm variance. This framework was essential in analyzing adverse event proportions, response rates, and survival outcomes, each of which exhibited different degrees of variability.

This meta-analysis advances the methodological rigor and clinical relevance of evidence synthesis in metastatic melanoma. While our central finding, namely, that combination immune checkpoint inhibitor therapy offers superior efficacy at the cost of increased toxicity, aligns with prior clinical trials, the principal contribution of our study lies in its integrative and comprehensive analytic framework. We jointly modeled treatment efficacy and adverse events across heterogeneous trials using an FEM, an REM, and a GLMM, thereby enhancing generalizability beyond the constraints of individual studies. Notably, our application of time-resolved modeling strategies to OS and PFS, based on data extracted from Kaplan–Meier curves, represents a novel methodological contribution. This allowed us to model survival outcomes as continuous trajectories rather than rely solely on aggregated endpoints, offering a more nuanced picture of treatment benefit over time.

By aligning statistical techniques with the structural complexity of clinical outcomes, our approach bridges the gap between inference and decision-making in oncology. The application of GLMMs to logit-transformed survival proportions, with the explicit modeling of between-study variability, marks a significant innovation in the field. This framework, emphasizing transparency, flexibility, and robustness, not only strengthens the reliability of our conclusions but also provides a generalizable template for future meta-analyses where time-to-event outcomes and heterogeneous data sources are central. As immunotherapy continues to evolve, such methodologies can inform treatment selection, patient counseling, and the design of future clinical trials.

## Figures and Tables

**Table 1:** Pooled prevalence estimates of adverse events by therapy group, with heterogeneity indicators.

Adverse Event	Therapy Type	Study Arms	Patients	$I^{2}$(%) ^†^	Point Estimate	95% CI
At least one AE *	M	10	3222	99.53 ^g^	0.8194	(0.6562, 0.9152)
	C	6	1801	61.50 ^r^	0.9322	(0.9111, 0.9534)
Pneumonitis *	M	10	2650	31.99 ^r^	0.0195	(0.0126, 0.0265)
	C	6	1801	70.88 ^r^	0.0555	(0.0335, 0.0775)
Hypothyroidism *	M	10	2638	85.43 ^r^	0.1041	(0.0729, 0.1353)
	C	6	1801	47.72 ^r^	0.1878	(0.1597, 0.2160)
Hyperthyroidism *	M	7	2244	92.31 ^g^	0.0339	(0.0173, 0.0654)
	C	5	1707	81.59 ^r^	0.1409	(0.0983, 0.1836)
Colitis *	M	10	2558	80.44 ^r^	0.0300	(0.0161, 0.0439)
	C	6	1801	81.22 ^r^	0.0753	(0.0450, 0.1056)
Diarrhea *	M	9	2567	90.14 ^g^	0.1845	(0.1367, 0.2443)
	C	6	1801	88.67 ^r^	0.3457	(0.2734, 0.4180)
Increased AST *	M	3	1231	0.00 ^f^	0.0670	(0.0531, 0.0810)
	C	5	1487	89.74 ^r^	0.1732	(0.1078, 0.2385)
Increased ALT *	M	3	1231	0.00 ^f^	0.0738	(0.0592, 0.0884)
	C	5	1487	88.64 ^r^	0.1881	(0.1226, 0.2536)

M—monotherapy; C—combination therapy. ^†^ Superscript indicates model type used: ^f^ FEM, ^r^ REM, ^g^ GLMM. * Statistically significant difference between two therapy types at p<0.001.

**Table 2:** Comparison of clinical benefit outcomes between monotherapy and combination therapy, with pooled prevalence and heterogeneity indicators.

Outcome	Therapy Type	Study Arms	Patients	$I^{2}$ (%) ^†^	Point Estimate	95% CI
CR *	M	7	2458	91.46 ^g^	0.1251	(0.0858, 0.1791)
	C	5	885	59.29 ^r^	0.1749	(0.1348, 0.2151)
PR *	M	8	2504	79.49 ^r^	0.2029	(0.1663, 0.2394)
	C	5	885	0.00 ^f^	0.3448	(0.3135, 0.3761)
OR *	M	8	2504	94.31 ^g^	0.3156	(0.2391, 0.4037)
	C	5	885	64.94 ^r^	0.5224	(0.4652, 0.5796)
SD *	M	8	2504	91.73 ^g^	0.1676	(0.1257, 0.2199)
	C	5	885	78.09 ^r^	0.1007	(0.0589, 0.1425)

M—monotherapy; C—combination therapy. ^†^ Superscript indicates model type used: ^f^ FEM, ^r^ REM, ^g^ GLMM. * Statistically significant difference between monotherapy and combination therapy groups at p<0.001. CR = complete response; PR = partial response; OR = overall response; SD = stable disease.

**Table 3:** GLMM estimates for logit-transformed OS, including time, therapy type, and their interaction.

Fixed Effect	Estimate	Std. Error	z-Value	*p*-Value
Intercept	1.089	0.146	7.471	0.000
Therapy: Monotherapy	−0.639	0.162	−3.951	0.000
Time (Months)	−0.014	0.003	−4.552	0.000
Interaction: Time × Monotherapy	−0.004	0.004	−1.143	0.253

**Table 4:** Model-based predictions of OS at selected time points with 95% confidence intervals for monotherapy and combination therapy.

Time (Months)	Therapy Type	Predicted OS (%)	95% CI Lower	95% CI Upper
12	C	71.5	66.2	76.2
24	C	67.8	62.8	72.5
36	C	64.0	58.7	68.9
48	C	59.9	53.9	65.7
60	C	55.7	48.6	62.7
12	M	55.7	52.0	59.4
24	M	50.3	47.0	53.6
36	M	44.8	41.7	47.9
48	M	39.4	36.4	42.5
60	M	34.3	31.1	37.6

M—monotherapy; C—combination therapy.

**Table 5:** GLMM estimates for logit-transformed PFS, including time, therapy type, and their interaction.

Fixed Effect	Estimate	Std. Error	z-Value	*p*-Value
Intercept	0.005	0.220	0.024	0.981
Therapy: Monotherapy	−0.776	0.266	−2.920	0.003
Time (Months)	−0.008	0.003	−2.319	0.020
Interaction: Time × Monotherapy	−0.006	0.004	−1.535	0.125

**Table 6:** Model-based predictions of PFS at selected time points with 95% confidence intervals for monotherapy and combination therapy.

Time (Months)	Therapy Type	PFS (%)	95% CI Lower	95% CI Upper
12	C	47.9	37.9	58.0
24	C	45.6	36.1	55.5
36	C	43.4	33.9	53.3
48	C	41.2	31.6	51.5
60	C	39.0	29.0	50.0
12	M	28.3	23.0	34.2
24	M	25.2	20.4	30.6
36	M	22.3	18.0	27.3
48	M	19.6	15.7	24.3
60	M	17.2	13.5	21.7

M—monotherapy; C—combination therapy.

## Data Availability

The data supporting the findings of this study were extracted from published articles included in the meta-analysis. The full list of references is listed in Appendix tables, and extracted outcome data are available from the authors upon reasonable request.

## Abbreviations

The following abbreviations are used in this manuscript:

AE	Adverse Event
CI	Confidence Interval
CR	Complete Response
CTLA-4	Cytotoxic T-Lymphocyte-Associated Protein 4
DL	DerSimonian–Laird
FEM	Fixed-Effects Model
GLMM	Generalized Linear Mixed Model
ICI	Immune Checkpoint Inhibitor
irAE	Immune-Related Adverse Event
KM	Kaplan–Meier
OR	Overall Response
OS	Overall Survival
PD-1	Programmed Cell Death Protein 1
PFS	Progression-Free Survival
PR	Partial Response
REM	Random-Effects Model
SD	Stable Disease
SE	Standard Error

## Appendix A. Summary of Clinical Trials and Studies Included in the Analysis

**Table A1:** Summary of clinical trials and studies included in the analysis—Part 1.

Study Acronym	Ref.	Therapy	Population	Treatments	*N*
		Type			
CheckMate-067	[2]	M	Previously untreated patients with unresectable stage III or IV melanoma.	Nivolumab 3 mg/kg every 2 weeks.	316
		M		Ipilimumab 3 mg/kg every 3 weeks for four doses.	315
		C		Nivolumab 1 mg/kg plus ipilimumab 3 mg/kg every 3 weeks for four doses, followed by nivolumab 3 mg/kg every 2 weeks.	314
CheckMate-037	[34]	M	Previously treated patients with advanced melanoma who experienced disease progression while receiving treatment with ipilimumab.	Nivolumab 3 mg/kg every 2 weeks.	268
CheckMate-066	[4]	M	Previously untreated patients with wild-type BRAF advanced melanoma stage III or IV.	Nivolumab 3mg/kg every 2 weeks.	210
Keynote-006	[23]	M	Previously untreated patients with advanced melanoma stage III or IV.	Pembrolizumab 10 mg/kg once every 2 or 3 weeks.	556
		M		Ipilimumab 3 mg/kg once every 3 weeks for four cycles.	278
CheckMate-204	[24]	C	Previously treated patients with asymptomatic melanoma brain metastases, phase II clinical trial.	Nivolumab 1 mg/kg plus ipilimumab 3 mg/kg every 3 weeks for four doses, followed by nivolumab 3 mg/kg every 2 weeks.	119
CheckMate-511	[25]	C	Previously untreated patients with advanced melanoma stage III or IV.	Nivolumab 3 mg/kg plus ipilimumab 1 mg/kg once every 3 weeks for four doses.	180
		C		Nivolumab 1mg/kg plus ipilimumab 3 mg/kg once every 3 weeks for four doses.	178
				After 6 weeks, all patients received NIVO 480 mg once every 4 weeks.	
Keynote-001	[26]	M	Previously treated or treatment-naive patients with advanced melanoma, phase Ib clinical trial.	Pembrolizumab 2 mg/kg every 3 weeks, 10 mg/kg every 3 weeks, or 10 mg/kg every 2 weeks.	655

M—monotherapy; C—combination therapy.

**Table A2:** Summary of clinical trials and studies included in the analysis—Part 2.

Study Acronym	Ref.	Therapy	Population	Treatments	*N*
		Type			
Keynote-002	[27]	M	Previously treated patients with advanced melanoma, phase II study clinical trial.	Pembrolizumab 2 mg/kg every 3 weeks.	178
		M		Pembrolizumab 10 mg/kg every 3 weeks.	179
CheckMate-069	[28]	C	Previously untreated patients with advanced melanoma stage, phase II study clinical trial.	Nivolumab 1 mg/kg plus ipilimumab 3 mg/kg every 3 weeks for 4 doses, followed by nivolumab 3 mg/kg every 2 weeks.	94
		M		Ipilimumab 3 mg/kg plus placebo following the same schedule.	46
Hribernik 2020	[8]	M	Previously untreated patients with advanced or metastatic melanoma stage III or IV, retrospective study.	Pembrolizumab 2 mg/kg every 3 weeks and fixed dose of 200 mg every 3 weeks.	138
Hribernik 2022	[29]	M	Previously untreated patients with advanced or metastatic melanoma stage III or IV, a pilot study.	Anti-PD-1 or anti-CTLA-4 ICI. The specific therapeutic protocol is not reported.	58
Hribernik 2024	[30]	M	Previously untreated patients with advanced or metastatic melanoma stage III or IV, a prospective study.	Anti-PD-1 or anti-CTLA-4 ICI. The specific therapeutic protocol is not reported.	71
CheckMate-915	[31]	M	Previously untreated patients with stage IIIB-D or IV advanced melanoma.	Nivolumab 480 mg every 4 weeks.	917
		C		Nivolumab 240 mg every 2 weeks plus ipilimumab 1 mg/kg every 6 weeks.	916
CheckMate-238	[32] and [33]	M	Previously untreated patients with stage III or IV advanced melanoma, phase III study clinical trial.	Nivolumab 3 mg/kg every 2 weeks.	452
		M		Ipilimumab 10 mg/kg every 3 weeks for 4 doses and then every 12 weeks.	453

M—monotherapy; C—combination therapy.
